# Freshwater unionid mussels threatened by predation of Round Goby (*Neogobius melanostomus*)

**DOI:** 10.1038/s41598-022-16385-y

**Published:** 2022-07-27

**Authors:** Kyle H. Clark, Deborah D. Iwanowicz, Luke R. Iwanowicz, Sara J. Mueller, Joshua M. Wisor, Casey Bradshaw-Wilson, William B. Schill, J. R. Stauffer, Elizabeth W. Boyer

**Affiliations:** 1 Department of Ecosystem Science & Management, Penn State University, University Park, PA USA; 2 U.S. Geological Survey, Eastern Ecological Science Center, Kearneysville, WV USA; 3 Pennsylvania Fish & Boat Commission, Bellefonte, PA USA; 4 Department of Environmental Science, Allegheny College, Meadville, PA USA

**Keywords:** Invasive species, Environmental impact

## Abstract

Indigenous freshwater mussels (Unionidae) are integral to riverine ecosystems, playing a pivotal role in aquatic food webs and providing ecological services. With populations on the decline worldwide, freshwater mussels are of conservation concern. In this study, we explore the propensity of the invasive Round Goby *(Neogobius melanostomus)* fish to prey upon indigenous freshwater mussels. First, we conducted lab experiments where Round Gobies were given the opportunity to feed on juvenile unionid mussels and macroinvertebrates, revealing rates and preferences of consumption. Several Round Gobies consumed whole freshwater mussels during these experiments, as confirmed by mussel counts and x-ray images of the fishes. Next, we investigated Round Gobies collected from stream habitats of the French Creek watershed, which is renowned for its unique and rich aquatic biodiversity. We developed a novel DNA metabarcoding method to identify the specific species of mussels consumed by Round Goby and provide a new database of DNA gene sequences for 25 indigenous unionid mussel species. Several of the fishes sampled had consumed indigenous mussels, including the Elktoe (non-endangered), Creeper (non-endangered), Long Solid (state endangered), and Rayed Bean (federally endangered) species. The invasive Round Goby poses a growing threat to unionid mussels, including species of conservation concern. The introduction of the invasive Round Goby to freshwaters of North America is shaping ecosystem transitions within the aquatic critical zone having widespread implications for conservation and management.

## Introduction

Freshwater mussels (Unionidae) are integral to aquatic food webs; and serve many ecological functions by consuming microbes, providing food, stabilizing sediments, cycling nutrients, filtering water, and enhancing water quality^1–3^. Populations of indigenous freshwater mussels have been declining worldwide for decades and are a focus of widespread conservation and management efforts^4,5^. Many interacting factors can contribute to the decline of unionid mussel populations, including modifications of stream corridors (e.g., via dams, channelization, and dredging), habitat alteration, harvesting, climate change, water quality degradation, decreasing fish host populations, and aquatic invasive species^2,6,7^.

Invasive species are among the greatest threats to aquatic biodiversity worldwide, causing negative ecological, economic, and health impacts^8,9^. This study considers potential effects of the invasive Round Goby *(Neogobius melanostomus)* on indigenous species of freshwater mussels. The Round Goby is a benthic fish that originated in the Caspian Sea region of Eurasia^10^ and has become an invasive species of significant concern in North America and other parts of the world. This species was first discovered in North America in 1990 in the province of Ontario’s St. Clair River. They are likely to have been translocated in ballast water from cargo ships^10,11^, a hypothesis that has been recently bolstered by a critical study of the Round Goby’s population genetics^12^. Round Gobies are tiny and aggressive fish that can outcompete indigenous species for food and spawning habitat^13,14^. In the three decades since their introduction to North America, they have rapidly expanded in range. Of the recent fish introductions into the Laurentian Great Lakes watershed, Round Gobies have been the most prolific and widespread^15^. The Round Goby is currently among the most abundant fish species in the Great Lakes and their watersheds, where it has caused many ecosystem changes^11,14,16^.

**Figure 1:**
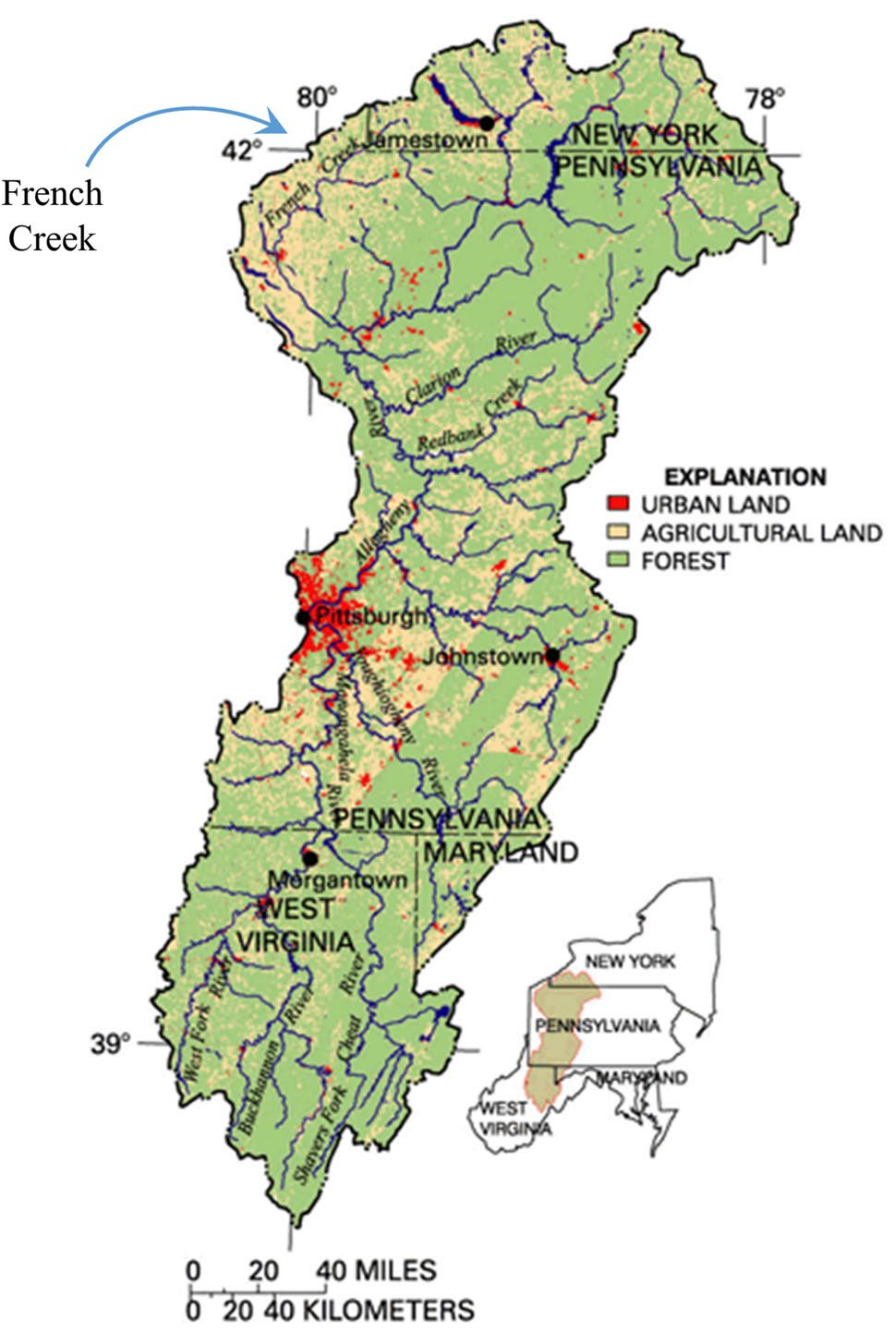
The Allegheny and Monongahela River Basins (49,585 km^2^) of the states of New York, Pennsylvania, West Virginia, and Maryland, USA. The invasive Round Goby has recently been introduced to the headwaters of this basin within the French Creek watershed. Figure adapted from USGS, with north direction facing upward ^21^.

Despite public education and natural resources management efforts aiming to prevent further spread, the Round Goby has recently expanded its range over the watershed divide of the Great Lakes basin into the Allegheny River basin of New York and Pennsylvania, USA (Fig. 1). Our research group is in the unique scientific situation of having pinpointed the recent introduction of the invasive Round Goby in this basin’s headwaters, having studied fish and mussel populations before and after its introduction. This presents a research opportunity to carefully observe the dispersal of the Round Goby as it continues its expansion throughout the stream and river network and quantify its impacts on aquatic biodiversity and water quality. Within the Allegheny basin, the Round Goby first appeared in the French Creek watershedin 2013; and has since established populations in Lake LeBoeuf, the entire length of LeBoeuf Creek, and a section of the mainstem French Creek downstream of the confluence with LeBoeuf Creek^14,17–19^. These locations are further detailed in Clark et al.^19^.

The French Creek watershed drains an area of 3330 km^2^ and is situated in the headwaters of the Allegheny and Monongahela River Basins (see Fig. 1). The watershed is globally significant due to its unique and rich aquatic biodiversity, stemming from glaciation during the Pleistocene epoch and paleo-historical stream captures^13^. French Creek and its tributaries harbor a diverse assemblage of 80 fish species and 28 freshwater mussel species, widely recognized for their importance in biodiversity and conservation and viewed as a conservation refuge for species of unionid mussels^19,20^. The US Endangered Species Act represents a commitment to preserving species that enrich society. Species listed are endangered (in danger of becoming extinct) or threatened (likely to become endangered). Of particular interest in the French Creek watershed is the presence of 12 indigenous freshwater mussel species that are imperiled and are listed as state- or federal- endangered species. The four mussel species found in this watershed that are listed as endangered under the federal Endangered Species Act include the Clubshell (*Pleurobema clava*), Northern Riffleshell (*Epioblasma rangiana)*, Rayed Bean (*Paetulunio fabalis*), and Snuffbox (*Epioblasma triquetra*)^19^. The watershed is also home to 14 indigenous fish species of darters, some of which act as host species for unionid mussels and are listed as endangered, such as the Sand Darter (*Ammocrypta pellucida)*^19^.

The invasive Round Goby can directly affect mussel populations through predation and consumption of mussel species, and by serving as a suitable or unsuitable host for mussels. It is well known that the Round Goby consume invasive zebra mussels (*Dreissena polymorpha*) and invasive dreissenid quagga mussels (*Dreissena bugensis*) throughout the Great Lakes basin^22–24^. Our research group recently documented the consumption of indigenous unionid freshwater mussels by Round Goby in French Creek^18^, and questions remain about the propensity of Round Gobies to affect individual unionid species and larger mussel populations. Round Gobies are known as voracious feeders and, in addition to mussels, have been observed consuming additional food sources such as other bivalves, macroinvertebrates, fish embryos, and small fishes^14,15,18,25,26^. Round Gobies have had wide-ranging effects on various species in the Great Lakes basin through interspecific competition and predation^15^. They have directly competed for habitat and food with indigenous fish species, including darters (Percidae), sculpins (Cottidae), and catfish (Ictaluridae), which can act as hosts as part of the mussels’ lifecycle^14,15,19,27,28^.

Further, the invasive Round Goby can indirectly affect mussel populations through predation and displacement of fish species that serve as hosts for larval mussels. They can alter the specific indigenous host-fish communities required for freshwater mussel reproduction^14,15,19,27–29^. Freshwater mussels exhibit unique reproductive strategies that require aquatic host organisms to complete their life cycle^7^. Gametes from mussels combine and develop into glochidia, which attach to host organisms as they grow. While various mussel species can exploit many host organisms, some mussels are highly specialized and can only utilize a few or a single host species to complete their life cycle. For freshwater mussels to successfully reproduce, specific host fish must be present and in relatively great abundance. The host species for the entire rich diversity of freshwater mussel species found in the French Creek watershed were quantified and documented by Clark et al.^19^.

In this exploratory study, we consider the propensity of Round Goby to prey upon indigenous freshwater mussel species, thereby posing a potential threat to their populations. First, we conducted laboratory experiments where Round Gobies were given the opportunity to feed on juvenile unionid mussels and macroinvertebrates, revealing their rates of (and preferences for) mussel consumption. Next, we investigated Round Gobies collected from their (newly invaded) stream habitats in the French Creek watershed, using novel DNA metabarcoding methods to reveal the specific mussel species they consumed. We make data (e.g., new gene sequences of freshwater unionid mussel species) and results from this project available in public data repositories (see section on data availability).

## Results

### Lab studies reveal consumption of unionids by Round Goby

**Figure 2:**
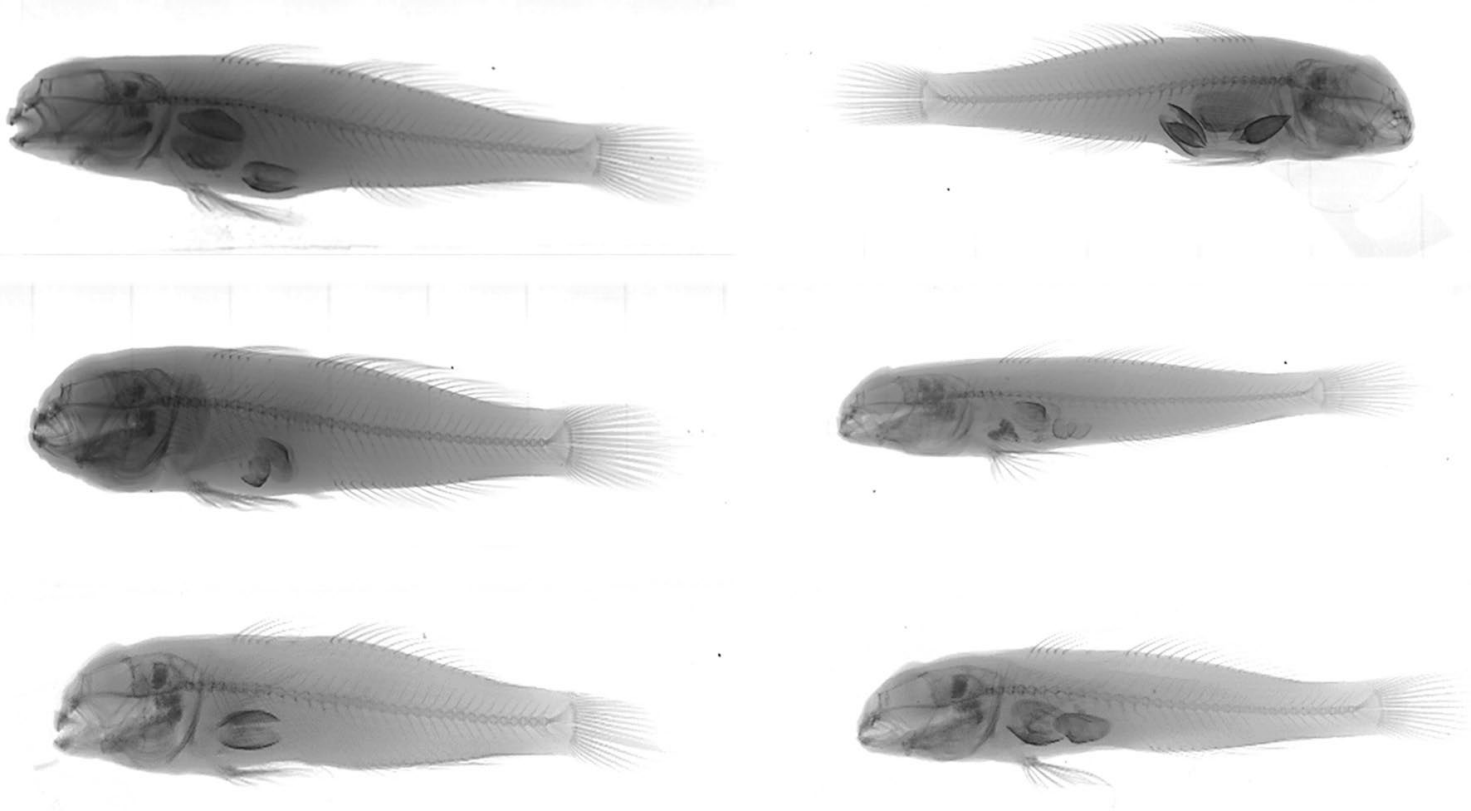
X-ray images show that 6 Round Gobies *(Neogobius melanostomus), * ranging in length from 65 to 103 mm, consumed whole juvenile unionid mussels. Image credit: Kyle Clark.

The laboratory stream table experiments highlight the potential for Round Goby to prey upon indigenous unionids. X-rays of the Round Gobies provided unequivocal evidence that six individual fishes (averaging 80 mm in length, ranging from 65 to 103 mm) had consumed juvenile Plain Pocketbook mussels *(Lampsilis cardium)* throughout the three experiments, given that whole mussels were found intact within them (Fig. 2). At the end of each experiment, the number of whole mussels found inside the Round Goby stomachs ranged from 1 to 4 (supporting Table S-1^30^). The average size of these whole mussels ranged from 3.5 to 9.0 mm in length, with an average of 6.8 mm.

In addition to the mussels that were swallowed whole and remained intact while digested by Round Goby, results also revealed that some mussels from the stream tables were missing at the end of each experiment (supporting Table S-1^30^). We hypothesize that the Round Gobies consumed the missing mussels, which were likely crushed, consumed, and excreted. Previous studies have shown that Round Goby can crush bivalve shells with their upper and lower molariform teeth and consume Zebra mussels efficiently by swallowing them whole or crushing them^22,23,31^. We observed shell fragments amidst the sediments on the stream tables, providing qualitative evidence supporting this notion.

Further, some whole dead mussels were found after each experiment (supporting Table S-1^30^), lying ajar on the surface of the stream substrate. It is likely that most dead mussels were first consumed whole by the gobies and subsequently excreted, though some natural mortality also can occur. Based on our observations, we calculated mortality rates for each experiment as the sum of freshwater mussels that were not recovered (those consumed whole and found intact, and those presumed consumed and found dead or missing). Freshwater mussel mortality was higher in experiments with Round Gobies than those without Round Gobies. In experiment 1, control stream tables 3 and 4 had no Round Gobies present, with 2.1% mortality of mussels (7/330) observed, revealing some mussels’ mortality that is not attributed to predation. For the experiments with Round Goby present, the mussel mortality rates observed were higher, with 12.1% in experiment 1 (40/330), 10.5% in experiment 2 (42/400), and 9.5% in experiment 3 (38/400).

### DNA metabarcoding reveals unionid species consumed by Round Goby

Round Gobies are known to prey on mussels, but species identification of crushed shells or only soft tissue in stomachs can be laborious and sometimes even impossible. In cases where mussel hard shells are unidentifiable to the species level based on morphology or only soft tissue is present in a stomach sample, environmental organismal DNA (eDNA) offers a means of mussel identification. Because of the imperiled status of unionid mussels in French Creek and their wide faunal diversity, mussels are ideal candidates for applying eDNA detection methods to determine the species of mussels upon which Round Gobies are foraging. We sampled 39 Round Gobies directly from their (newly invaded) stream habitats within the French Creek watershed. We acknowledge the limitation of basing our analysis on this sample representing a single snapshot in time, as samples taken at other seasons or other times of day could show different results. Nonetheless, our collection of Round Gobies for this study allows exploration of their diet, seeking to understand if any of the specimens had consumed freshwater mussels. Further, for 25 species of indigenous unionid mussels found in the watershed (Table 1), we developed a multispecies genetic marker panel providing species-specific “fingerprints” to reveal which unionid mussel species the Round Gobies may have consumed as part of their diet. Within the French Creek watershed, these 25 freshwater mussel species represent ~ 90% of the historical mussel biodiversity (representing 25 of 28 species documented to date in historical records, though 2 species are likely no longer present) and ~ 96% of the contemporary biodiversity (representing 25 of 26 mussel species currently thought to be present).

**Table 1:** Target unionid mussel species in the French Creek watershed, USA, their conservation status, and primer combinations﻿.

Common name	Scientific name	NCBI GenBank accession#	Conservation concern status	Forward primer	Reverse primer
Plain pocketbook	*Lampsilis cardium*	AF120653	OK	UASFWD2	UASREV16
Elktoe	*Alasmidonta marginata*	AF156502	OK	UASFWD2	UASREV6
Creek heelsplitter	*Lasmigona compressa*	AF156503	State proposed endangered	UASFWD7	UASREV15
Spike	*Eurynia dilatata*	AF156507	OK	UASFWD4	UASREV19
Wavy-rayed lampmussel	*Lampsilis fasciola*	AF156520	OK	UASFWD2	UASREV16
Rainbow mussel	*Cambarunio iris*	AF156524	State proposed endangered	UASFWD2	UASREV13
Snuffbox	*Epioblasma triquetra*	AF156528	Federally listed endangered	UASFWD2	UASREV14
Clubshell	*Pleurobema clava*	AF231754	Federally listed endangered	UASFWD2	UASREV18
Long-solid	*Fusconaia subrotunda*	AY613824	State proposed endangered	UASFWD2	UASREV20
Rabbitsfoot	*Theliderma cylindrica*	ON148513	State listed endangered	UASFWD4	UASREV17
Rayed bean	*Paetulunio fabalis*	DQ220726	Federally listed endangered	UASFWD10	UASREV12
Round pigtoe	*Pleurobema sintoxia*	EF033253	State proposed endangered	UASFWD2	UASREV21
Pocketbook	*Lampsilis ovata*	EF033262	OK	UASFWD2	UASREV16
Mucket	*Ortmanniana ligamentina*	EF033300	OK	UASFWD8	UASREV10
White heelsplitter	*Lasmigona complanata*	HM849078	State proposed endangered	UASFWD6	UASREV15
Paper pondshell	*Utterbackia imbecillis*	HM856637	OK	UASFWD3	UASREV7
Black sandshell	*Ligumia recta*	KC291717	OK	UASFWD2	UASREV11
Giant floater	*Pyganodon grandis*	MF544440	OK	UASFWD2	UASREV8
Cylindrical papershell	*Anodontoides ferussacianus*	MG199637	State proposed endangered	UASFWD9	UASREV6
Fatmucket	*Lampsilis siliquoidea*	MH012239	OK	UASFWD2	UASREV2
Fluted-shell	*Lasmigona costata*	MH012240	OK	UASFWD7	UASREV15
Three-ridge	*Amblema plicata*	MH633633	State proposed threatened	UASFWD3	UASREV1
Northern riffleshell	*Epioblasma rangiana*	MK044909	Federally listed endangered	UASFWD2	UASREV14
Kidneyshell	*Ptychobranchus fasciolaris*	MK044964	OK	UASFWD2	UASREV4
Creeper	*Strophitus undulatus*	MK308227	OK	UASFWD4	UASREV4

Conservation concern status for mussel listings at the state level refers to the state of Pennsylvania, for the federal level refers to USA, and a status of “OK” indicates that the mussel species is not currently considered to be endangered in Pennsylvania.

**Figure 3:**
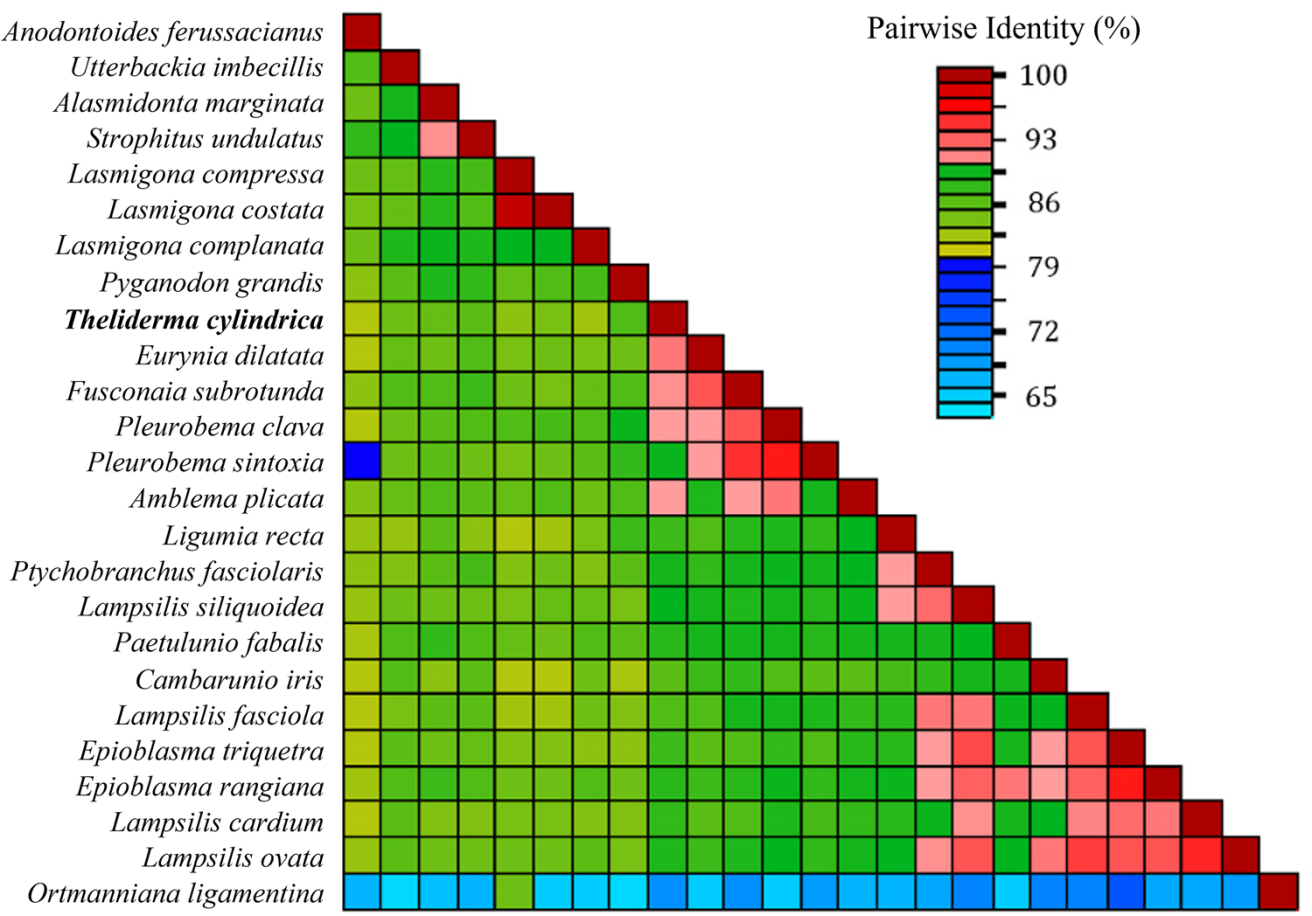
This pairwise identity matrix is based on the 278 bp of the cytochrome c oxidase subunit 1 gene (COI) used in the reference database for 25 target unionid mussel species that were used for sequence read mapping. It includes the *T. cylindrica* COI sequence identified in this study. Sequence identities ranged from 63 to 99% across these 278 nucleotides.

We developed custom, specific primers for the Cytochrome C Oxidase Subunit 1 (COI) gene to produce amplicon libraries for DNA sequencing. This COI gene is the standard gene region for barcoding animal groups and is a primary gene used with the Barcode of Life reference database of public genetic sequence collections from the National Center for Biotechnology Information (NCBI 2022). We established COI sequences for 25 unionid mussel species (supporting Dataset S-1^30^). Rolling circle amplification and sequencing of *T. cylindrica* and *L. complanata* led to identifying a partial sequence (668 bp) of the COI gene (supporting Datasets S-1 and S-2^30^). We have obtained a GenBank accession number for the partial sequences (see Table 1), but we could not recover full mitochondrial genomes due to evident DNA degradation in both samples. We successfully designed 26 primers for the amplification of the target unionid mussel species for this project (supporting Table S-2^30^). These primers were used to generate a lab-tailored degenerate primer mix to amplify COI. This primer set successfully amplified a single product in control mussel samples. Those products were sequenced and were 99–100% identical to those in GenBank. The few nucleotide differences likely represent uncharacterized differences across haplotypes. These primer sets did not amplify spurious non-specific products using goby DNA as a template. Other than nucleotide similarity between *L. compressa* (AF156503) and *L. costata* (MH012240; 99%), sequence identities ranged from 63 to 96% across these 278 nucleotides (Fig. 3).

Metabarcoding results provide strong evidence that some of the Round Gobies sampled from French Creek tributaries had consumed freshwater mussels. We found that 2 of the 39 fishes, or 5.1% tested positive for unionid DNA in their stomach tissue (supporting Table S-3^30^). Using the DNA metabarcoding procedure, we identified four species of unionids in the stomach contents. The individual Round Gobies that preyed upon mussels consumed the Elktoe mussel (*Alasmidonta marginata*) in great relative abundance as well as the Creeper mussel *(Strophitus undulatus),* the Pennsylvania proposed endangered Long Solid mussel (*Fusconaia subrotunda)*, and the federally endangered Rayed Bean mussel (*Paetulunio fabalis*) (Table 2). Relative abundance is defined as the proportion of species-specific mapped reads, relative to all reads mapped to unionid sequences in our reference list of species inhabiting French Creek.

**Table 2:** Relative abundance of mapped COI sequences of indigenous unionid mussels from two Round Goby fishes (*N. melanostomus* samples #RG26 and RG28, collected from French Creek, Pennsylvania) with unionid DNA present in their stomach contents.

	*A.marginata* Elktoe mussel	*F. subrotunda* Long Solid mussel	*S. undulatus* Creeper mussel	*P. fabalis* Rayed Bean mussel
*N. melanostomus* (RG26)	99%	1%	< 1%	< 1%
*N. melanostomus* (RG28)	93%	< 1%	< 1%	7%

## Discussion

Invasive species can pose significant threats to freshwater aquatic biodiversity. This lab-based study shows that Round Goby predation has the propensity to contribute to the loss of indigenous unionid mussels through direct consumption. Poos et al.^32^ suggested mussel species suspected of endangerment might be potential prey of the Round Goby, which was first documented by our research group in a study where we looked at stomach contents of Round Goby to determine their diet^18^. Our laboratory feeding experiments and x-ray imaging provided further evidence that Round Gobies from French Creek prey on juvenile unionid mussels through direct consumption. Further, the use of data collected in situ in this study affirms our laboratory studies’ results through the analysis of DNA from fish stomachs. We developed a novel DNA metabarcoding method that is the first study to identify the specific species of unionid mussels that Round Goby is consuming. Our results show that several, but not all, of the individual Round Gobies that we sampled from the French Creek watershed had consumed unionid mussels—including the non-endangered Elktoe, the non-endangered Creeper, the Pennsylvania proposed endangered Long Solid, and the federally endangered Rayed Bean species.

Selective size predation is one of many factors potentially affecting the consumption of mussel species. Previous laboratory studies of dreissenid mussels^23^ and unionid mussels^18^ showed that Round Gobies of small lengths (e.g., 30–44 mm) consumed significantly more mussels than larger fishes and showed that most Round Gobies swallow whole bivalves rather than using their teeth to consume parts. Bradshaw-Wilson^18^ showed that as the length of Round Gobies increased (e.g., > 75 mm), their diets shifted to sphaeriids. In our laboratory study, the Round Gobies ranged from 40 to 103 mm with an average of 60 mm, and the juvenile unionid Plain Pocketbook Mussels consumed by Round Gobies ranged from 3.5 to 9 mm in length. While many unionids in the French Creek watershed are large in size (with most adult species ranging in size from 40 to188 mm in length), some smaller species and the juveniles of all species are particularly vulnerable to predation by Round Goby. Based on the life cycle and reproductive timing of freshwater mussels in the French Creek watershed, juvenile unionid mussels small enough for consumption may be available only during specific periods of each year, as was the case for dreissenid mussels in the Great Lakes^31^.﻿

Previous work has demonstrated the importance of non-native predators in unionid ecology and conservation. For example, several known predators other than the Round Goby have been widely introduced beyond their native ranges that have affected unionid mussel populations in other regions of the world. These include muskrats in Europe^33^, the black carp in North America^34^, and non-native crayfish in North America and Europe^35^. Important questions remain about how populations of unionid mussel species and their host fish species will fare as invasive Round Goby populations increase and expand their range in the stream and river network. While our results indicate that Round Goby populations pose a potential threat to freshwater unionid mussels given their propensity to consume some individuals, we cannot conclude their impacts on freshwater mussel populations from this study alone. The effects of Round Goby predation depend on the complex factors affecting predation rates in nature and demographic controls on unionid populations, about which there is still much to be learned^6^. There is a pressing need for continued field-based observations of the Round Goby, including measurements of population sizes, range expansion, and rates of spread. This, in turn, contributes to the scientific basis needed to guide education, management, and engineering efforts that could help control the further spread of Round Goby. The metabarcoding methods pioneered in this study to identify specific species of mussels consumed, in whole or part, by predators will be helpful in follow-on and related assessments. Toward that goal, we provide (as supplemental information) our new database of DNA extracted from 25 species of indigenous unionid mussels that are found in the northeastern USA, including forward and reverse amplification primers and trimmed reference COI gene sequences.

The introduction of the invasive Round Goby to freshwaters of North America is significant, shaping ecosystem transitions within the aquatic critical zone that have widespread implications for the conservation and management of benthic fauna. Further research is needed to observe and understand how indigenous unionid communities will be shaped by the invasive Round Goby, along with alterations of their host fish populations, habitat change, climate change, overharvesting, and other stressors. In the aquatic critical zone, it is well documented that changes may alter the composition and function of their resident aquatic life, but less is known about how this, in turn, may have disproportionate impacts on neighboring human populations that depend on them^36^. Research challenges remain in predicting the complex interactions of interacting species, given nonlinear dynamics and feedbacks that can produce sudden changes or unexpected shifts when ecosystems exceed tipping point thresholds^37,38^. Emerging biotechnologies hold promise for controlling or eradicating unwanted introduced species; but carry risks of unanticipated consequences and uncertainty in how ecosystems will respond to control^39^.

## Methods

Our research involved work with animal subjects (unionid mussels and Round Goby fishes) and was conducted following relevant regulations and standard procedures. The field collections were carried out under Pennsylvania Fish and Boat Commission permits (# 2018-01-0136 and 2019-01-0026). The experimental protocols were approved by Penn State University’s Institutional Animal Care and Use Committee (IACUC# 201646941 and 201646962**)**. All new DNA sequencing data are made publicly available in GenBank (with accession numbers provided in Table 1) and a BioProject (# PRJNA813547) of the National Center for Biotechnology Information^40^.

### Propensity of Round Goby to consume unionid mussels in a controlled lab setting

#### Stream table setup

We conducted lab experiments to observe the potential predation of juvenile freshwater mussels by the Round Goby, following standard research protocols for work with animal subjects (IACUC# 201646962, Penn State University). We constructed four artificial stream tables in an aquatic laboratory, each measuring 3 × 2 m and featuring two run and two pool sections (each 0.63 × 0.56 × 0.46 m). Water flow was produced using eight Homsay 920 GPH submersible water pumps, which pumped water from a central reservoir tub into each table at the start of each run section. The water flow direction was clockwise for stream tables 1 and 3, and counterclockwise for stream tables 2 and 4. Water pumped into the stream tables exited via two drains located medially of each run section, where it flowed back to the central reservoir tub. Each stream table was filled with a 6 mm layer of substrate consisting of a mixture of sand, gravel (4–6 mm), and crushed stone (size 2B, with an average size of ~ 19 mm). The day before each experiment, field technicians traveled to local streams and collected macroinvertebrates using one minute D-frame kick net samples for each of the four stream tables. The macroinvertebrates and associated substrate were transported back to the facility and were introduced into each stream table system.

#### Preferential feeding experiments

Before each experiment commenced, juvenile Plain Pocketbook mussels (*Lampsillis cardium*) were introduced into each stream table (with 165 mussel specimens for experiment 1 and 100 mussels for experiments 2 and 3). This  widespread and abundant species is not imperiled in Pennsylvania, and mussels were provided for this study by the White Sulphur Springs National Fish Hatchery located in southeast West Virgina. The mussels were allowed to acclimate in the stream tables for 2 h before commencing each experiment. Ten Round Gobies were introduced into each stream system (stream tables 1 and 2 for experiment 1, and all tables for experiments 2 and 3). The total length (from nose tip to caudal tip) of each fish was measured prior to introduction and after the termination of experiments 2 and 3. Experiment 1 was conducted for 3 weeks, while experiments 2 and 3 were conducted for 8 days. During these experiments, Round Gobies were allowed to exist in the systems and feed preferentially, on the mussels and macroinvertebrates, for the allotted time before each investigation concluded. We acknowledge that in these experiments, the mussel abundances are higher and macroinvertebrate densities lower and less rich than commonly occur in the natural stream environment. Further, the Round Goby fish densities used are much higher than currently in the French Creek watershed, though are comparable to what is currently seen in parts of the Great Lakes basin. Nonetheless, the experiment scenarios allowed us to observe if Round Gobies would consume the mussels when given the choice to feed on a variety of food items.

#### Evaluation of unionids consumed by fish

Round Gobies were removed from the stream tables upon completion of each experiment. They were euthanized using > 250 mg/L buffered (pH ~ 7) tricaine-S (MS222) solution. The fish were submerged for 10 min beyond the cessation of opercular movement to ensure proper euthanasia, and tissues were collected after we confirmed complete euthanasia—compliant with AVMA guidelines and approved by the IACUC protocol. The Round Gobies were placed in a 10% solution of formalin for preservation, and after 2 weeks, they were rinsed with clean water and were placed in 70% ethanol for long-term storage. After fish were removed from the system, the water was drained, and the substrate was sifted to recover the remaining mussels. Mussels were counted, and live individuals were returned to holding tanks for use in subsequent experiments. To further assess whether Round Gobies had consumed mussels during the investigation, Round Gobies were x-rayed using a Bruker Skyscan 1176 micro-CT scanner. After that, the stomachs of each fish were excised, and the contents examined using a Leica CME dissection scope to confirm the identity of Plain Pocketbook mussels. Contents posterior to the stomach were not analyzed because they could not be reliably counted and identified.

### DNA metabarcoding to identify mussel species consumed by Round Goby in a stream setting

#### Fish and mussel sample acquisition

We collected 39 Round Gobies directly from streams within the French Creek watershed—their newly invaded natural stream habitats—in June 2018. We aimed to quantify which species, if any, of unionid mussels they consumed. Fish collection locations included LeBoeuf Creek at Moore Road and 100 m below the confluence of French Creek and LeBoeuf Creek. The unionid mussel populations and the environmental field settings at these locations are detailed by Clark et al.^19^. A team of field technicians collected fish by kick seining (3 m × 1 m × 9.5 mm nylon mesh) while moving downstream. Seining was the sampling method of choice compared to electrofishing to avoid possible regurgitation of food items prior to excision of fishes’ stomachs. The stream reaches sampled at each location were between 100 and 200 m in length and included riffle, run, and pool habitats. In addition to fish samples, unionid mussel samples from French Creek were also collected for analysis (under Pennsylvania Fish & Boat Commission collectors permits # 2018-01-0136 and 2019-01-0026). Following standard research protocols (under IACUC# 201646941, Penn State University), the Round Gobies collected were euthanized using buffered Tricaine-S (MS222) solution; and stomachs were excised using sterilized utensils before being placed in sterilized tubes filled with 97% ethanol. After excision of stomachs, fishes were placed in a 10% formalin solution for preservation. After 2 weeks, fishes were rinsed with clean water and transferred to 70% ethanol for storage. The stomach samples were immediately placed in ethanol and on ice in the field. Samples were stored in a freezer before being shipped to the US Geological Survey’s Eastern Ecological Science Center for various molecular ecology analyses. Once the fish and mussel samples arrived at this lab, they were recorded and stored at four °C until analysis.

#### Primer development

Specific primers targeting a moderately conserved region of the mitochondrial COI gene for 25 species of unionids inhabiting French Creek were designed. Previously a PCR-based amplification method utilizing restriction enzyme digests was used to identify genetic fingerprints of 25 unionid species inhabiting French Creek^41^. Here, we designed a new degenerate PCR primer set modified with sequencing overhangs to facilitate compatibility with a MiSeq amplicon sequencing method previously designed for 16S Amplicon sequencing. We targeted the locus of the mitochondrial COI gene of unionids known to inhabit the Atlantic Slope Drainage. Consensus sequences were derived using Multalin analysis and a tiling method to identify conserved primer binding regions flanking an ~ 300 bp region of the COI gene. This gene was targeted in part due to the availability of partial or complete sequences representing these target species in the NCBI reference database^40^. Cytochrome oxidase sequences were downloaded for the 25 unionid mussel species of interest. However, a COI sequence for the Rabbitsfoot (*Theliderma cylindrica*) mussel was absent from the NCBI database, which required us to sequence this region for an in-house reference (which is described later in the paper). We designed a degenerate primer cocktail specific to all mussel species of interest that amplified a ~ 289 bp product, with forward and reverse primers used for the amplification of unionid specific COI presented as supplemental information (see Table S-2^30^. We evaluated the suitability of the primers using samples from field identified mussels. For primer optimization, PCR was run across a gradient of annealing temperatures to determine suitability. In addition, we used Round Goby DNA as a template to evaluate specificity. In addition to Round Goby stomach samples, mussel samples of several species collected from French Creek were included as positive controls.

#### DNA extraction from tissue samples

Following the manufacturer’s protocols, tissue samples (including fish stomach and mussel tissue) were extracted with the Zymo Research ZymoBIOMICS 96 MagBead DNA Kit (San Diego, CA). Random samples of DNA extracts were analyzed on an Agilent 2100 Bioanalyzer using a high-sensitivity assay kit. Fragments in the target amplicon range were apparent (albeit not known to be of mussel origin). All samples were stored at − 20 °C until PCR was performed. DNA from both the *T. cylindrica* and *L. complanata* samples were analyzed for DNA quality.

#### Rolling circle amplification of mitochondrial genomes

To acquire COI sequences for *T. cylindrica* and *L. complanata,* we subjected archived DNA samples to rolling circle amplification (RCA) followed by amplicon sequencing on the MiSeq. In short, 2 µl of DNA template was added to 2 µl Equiphi29 DNA polymerase reaction buffer containing 1 µl of Exonuclease-resistant random primers (ThermoFisher). Samples were denatured by heating to 95 °C for 3 min followed immediately by cooling on ice for more than 5 min. A volume of 5 µl was added to an RCA master mix containing 1.5 µl of 10 × Equiphi29 DNA polymerase reaction buffer, 0.2 µl of 100 mM dithiothreitol, 8 µl of 2.5 mM dNTPs, 1 µl of Eqiphi29 DNA polymerase (10U) and 4.3 µl of nuclease-free water. The samples were heated to 45 °C for 3 h and then 65 °C for 10 min. Samples were then placed in ice and then frozen at − 20 °C. All RCA products were normalized to 0.2 ng/µl in 10 mM Tris–HCl, pH 8.5. Normalized RCA product was utilized as a template for an Illumina Nextera XT library preparation. Sequencing libraries were prepared following the Nextera XT Library Preparation Reference Guide (CT# 15031942 v01) using the Nextera XT Library Preparation Kit (Illumina, San Diego, CA). Final libraries were analyzed for size and quality using the Agilent BioAnalyzer with the accompanying DNA 1000 Kit (Agilent, Santa Clara, CA). Libraries were quantified using the Qubit H.S. Assay Kit (Invitrogen, Carlsbad, CA) and normalized to 4 nM using 10 mM Tris, pH 8.5. Libraries were pooled and run on the Illumina MiSeq at a concentration of 10 pM with a 5% PhiX spike with run parameters of 1 × 150. Bioinformatic processing of this data is outlined below.

#### Amplification of the cytochrome oxidase 1 gene

Extracted genomic DNA was used as template for end-point PCR. Samples evaluated were from mussels and round gobies (see supporting Table S-3^30^). The ~ 289 bp COI region was amplified with the mussel primers as follows. The amplification reaction contained 0.15 µM of each primer, 1 µL of the initial amplification product, and Promega Go Taq Green Master Mix following manufacturer recommendations for a 25 µL reaction. The thermocycler program consisted of an initial denaturing step of 95 °C for 3 min, followed by 30 cycles of 30 s at 95 °C, 30 s at 52 °C, and 1 min at 72 °C. Products were subjected to a final extension of 72 °C for 5 min then held until collection at 12 °C. An appropriately sized amplification product was confirmed for each reaction by electrophoresis of 5 µL of the reaction product through a 1.5% I.D. N.A. agarose gel (FMC Bioproducts) at 100 V for 45 min. PCR products were cleaned with the Qiagen Qiaquick PCR purification kit (Valencia, CA) and quantified using the Qubit dsDNA H.S. Assay Kit (Thermofisher Scientific, Grand Island, NY). Samples were diluted in 10 mM Tris buffer (pH 8.5) to a final concentration of 5 ng/µL.

#### Generation of mock mussel samples

To better understand and minimize sources of error or bias in the taxonomic assignment, we created a mock extraction by mixing sequences from known mussel taxa at defined concentrations. For each mussel, approximately 25-mg of tissue was extracted with the ZymoBIOMICS 96 MagBead DNA Kit (San Diego, CA) following the manufacturer’s protocol. The COI sequence was amplified from each species using the same primer-protocol combination described above. A total of 5 PCR products were mixed at equal concentration (mass/volume) to generate the mock sample (“Mock” hereafter). To confirm the identity of these inputs, each COI region was amplified and sequenced on the Illumina MiSeq during the same run as the Mock and samples.

#### Sequencing library preparation and quality assessment

Next-generation sequencing was performed on the Illumina MiSeq platform to observe species-specific sequences and determine the diet of the Round Goby. Inclusion of the overhangs on the amplification primers allowed us to utilize the Illumina 16S Metagenomic Sequencing Library Preparation protocol^42^. Amplicon libraries were prepared following the same manufacturer’s protocol. All samples were indexed using the Illumina Nextera XT multiplex library indices. DNA read size spectra were determined with the Agilent 2100 Bioanalyzer using the Agilent DNA 1000 Kit (Santa Clara, Calif.). Libraries were quantified with the Qubit dsDNA H.S. Assay Kit (ThermoFisher Scientific, Grand Island, N.Y.) and normalized to 4 nM (nM) using 10 mM (mM) Tris (hydroxymethyl) aminomethane buffer pH 8.5. A final concentration of 10 picomolar library with a 6.5% PhiX control spike was created with the combined pool of all indexed libraries. All bioinformatic operations were completed on CLC Genomic Workbench v20 (Qiagen, Valencia, Calif.).

#### Read filtering, trimming, and RNAseq metabarcoding assembly

FASTQ files from the sequencing runs were imported as paired-end reads into CLC Genomics Workbench v20.0.4 (Qiagen Bioinformatics, Redwood City, Calif.) for initial filtering of exogenous sequence adaptors and poor-quality base calls. The trimmed overlapping paired-end reads were mapped to the 25 target unionid sequences specific for the species of interest. Several mapping iterations were run using different levels of stringency. We utilized + 2/− 3 match-mismatch scoring and set the length fraction to 0.90. Analyses were iterated using different similarity fractions ranging from 0.90 to 0.99. Reads were annotated, and relative abundance was determined using a curated reference library (see supporting Datasets S-1 and S-2^30^).

## Data Availability

Supporting data and information for this manuscript^30^ are provided in the CUAHSI HydroShare digital data repository (which is supported by the U.S. National Science Foundation), at: http://www.hydroshare.org/resource/e46d4769a8a346fcaed7a27fcceb20ad. In addition to the supporting tables and datasets described herein, this resource includes additional photos (e.g., of indigenous unionid mussels and invasive Round Goby) from the study site. Metabarcoding (short reads) sequencing data are included in the supporting data resource, and also are deposited in NCBI BioProject ID: PRJNA813547 at https://www.ncbi.nlm.nih.gov/bioproject/?term=PRJNA813547.
